# A Comparison of the Adaptive Response of *Staphylococcus aureus* vs. *Streptococcus mutans* and the Development of Chlorhexidine Resistance

**DOI:** 10.3389/fmicb.2022.861890

**Published:** 2022-05-19

**Authors:** Marieke van de Lagemaat, Valerie Stockbroekx, Gésinda I. Geertsema-Doornbusch, Melissa Dijk, Vera Carniello, Willem Woudstra, Henny C. van der Mei, Henk J. Busscher, Yijin Ren

**Affiliations:** ^1^University of Groningen and University Medical Center Groningen, Department of Orthodontics, Groningen, Netherlands; ^2^University of Groningen and University Medical Center Groningen, Department of Biomedical Engineering, Groningen, Netherlands

**Keywords:** self-repair, antimicrobials, cell wall deformation, cell wall damage, solidification of cytoplasm, resistance

## Abstract

Antimicrobials with nonselective antibacterial efficacy such as chlorhexidine can be effective in reducing biofilm, but bear the risk of inducing resistance in specific bacteria. In clinical practice, bacteria such as *Staphylococcus aureus* have been found resistant to chlorhexidine, but other bacteria, including *Streptococcus mutans*, have largely remained susceptible to chlorhexidine despite its widespread use in oral healthcare. Here, we aim to forward a possible reason as to why *S. aureus* can acquire resistance against chlorhexidine, while *S. mutans* remains susceptible to chlorhexidine. Measurement of surface-enhanced fluorescence indicated that chlorhexidine caused gradual, but irreversible deformation to adhering green fluorescent *S. aureus* due to irreparable damage to the cell wall. Concurrently, the metabolic activity of adhering staphylococci was higher than of planktonic bacteria, suggesting efflux mechanisms may have been activated upon cell wall deformation, impeding the buildup of a high chlorhexidine concentration in the cytoplasm and therewith stimulating the development of chlorhexidine resistance in *S. aureus*. Exposure of *S. mutans* to chlorhexidine caused immediate, but reversible deformation in adhering streptococci, indicative of rapid self-repair of cell wall damage done by chlorhexidine. Due to cell wall self-repair, *S. mutans* will be unable to effectively reduce the chlorhexidine concentration in the cytoplasm causing solidification of the cytoplasm. In line, no increased metabolic activity was observed in *S. mutans* during exposure to chlorhexidine. Therewith, self-repair is suicidal and prevents the development of a chlorhexidine-resistant progeny in *S. mutans*.

## Introduction

The increasing resistance of bacteria to antimicrobials occurring over the past decades has become a major concern for public health (Howard et al., 2003; Roca et al., 2015; World Health Organization [WHO], 2018). Due to the increase in antimicrobial resistance among different bacterial strains, it is predicted that in 2050, antimicrobial-resistant infections will be the main cause of death (O’Neill, 2014). Chlorhexidine is a broad-spectrum antimicrobial and widely used in healthcare settings as a disinfectant for the skin, hands, and in oral healthcare products (Zhang et al., 2013; Okada et al., 2016). Chlorhexidine carries positively-charged groups that can bind to negatively-charged bacterial cell surfaces (Cieplik et al., 2019) to cause cell wall damage and catastrophic leakage of intracellular material. Mild cell wall damage and leakage of intracellular material will not cause immediate cell death. Instead, mild cell wall damage will yield loss of intracellular pressure, which is accompanied by minor cell wall deformation in adhering bacteria (Carniello et al., 2020). Bacterial cell wall deformation is only demonstrable using highly sensitive methods such as atomic force microscopy (Zuttion et al., 2020) or surface-enhanced fluorescence (Lee et al., 2011; Li et al., 2014; Carniello et al., 2018a). Severe, catastrophic cell wall damage will eventually result in bacterial death (Gilbert and Moore, 2005). These properties provide chlorhexidine with a broad-spectrum antimicrobial activity against both the Gram-positive and Gram-negative bacteria (Beyth et al., 2003). Chlorhexidine is considered safe for use in the oral cavity at appropriately low concentrations (Denton, 2001) and has become the “gold” standard in antibacterial mouthrinses (Balagopal and Arjunkumar, 2013).

Microbial resistance against chlorhexidine has long been considered rare if not impossible (Schlett et al., 2014; Saleem et al., 2016), but has recently been reported in *Staphylococcus aureus*, coagulase-negative staphylococci, *Klebsiella pneumoniae*, *Pseudomonas aeruginosa*, *Acinetobacter baumannii*, and *Candida albicans*. For these bacteria, the intensity of chlorhexidine use was found proportional with the development of resistance (Block and Furman, 2002). Importantly, after acquiring resistance to chlorhexidine, *Acinetobacter* spp., *K. pneumoniae*, and *Pseudomonas* spp. can develop cross-resistance to different antibiotics (Kampf, 2016). Horizontal gene transfer of chlorhexidine resistance at subminimal inhibitory concentrations of chlorhexidine has been reported in *Escherichia coli* (Jutkina et al., 2018). Exposure of *P. aeruginosa* to 4 μg/ml chlorhexidine caused downregulation of genes involved in membrane transport, oxidative phosphorylation, electron transport, and DNA repair, while multidrug efflux pump genes were upregulated (Nde et al., 2009).

Bacterial resistance against chlorhexidine in oral bacteria is still rare for reasons that have only been speculated upon. However, although chlorhexidine does not penetrate deeply in oral biofilms (Zaura-Arite et al., 2001), its prolonged substantive presence on oral soft tissues and low-level release (Reda et al., 2021) are ideal for developing resistance. Therefore, clinical use of chlorhexidine has been restricted to applications with clear patient benefits and elimination of its use in applications without healthcare benefits has been suggested (Kampf, 2016). While still being allowed for oral use, safeguarding the much-needed use of chlorhexidine for oral applications in absence of equally effective alternatives requires better understanding of why oral bacteria seem not to develop chlorhexidine resistance.

Therefore, we compare the response to chlorhexidine of *S. aureus*, a pathogen involved in a wide variety of clinical infections (McCormack et al., 2015) and *Streptococcus mutans*, a cariogenic oral pathogen (Loesche, 1986). First, nanoscopic deformation of the cell walls of both the strains, including a chlorhexidine-resistant *S. aureus* variant, was determined upon exposure to chlorhexidine using surface-enhanced fluorescence. Cell wall damage at the more microscopic, membrane level was studied after propidium iodide staining of chlorhexidine-exposed bacteria using fluorescence microscopy. Since maintenance of antimicrobial resistance is known to be energy-consuming, metabolic activity was monitored using 3-(4,5-dimethylthiazol-2-yl)-2,5-diphenyltetrazolium bromide (MTT) reduction assay during bacterial exposure to chlorhexidine. Based on the collective data obtained, a possible reason is forwarded through which *S. aureus* can readily develop resistance against chlorhexidine, while *S. mutans* remains susceptible to chlorhexidine.

## Materials and Methods

### Bacterial Strains, Growth Conditions, and Harvesting

In order to allow measurement of cell wall deformation using surface-enhanced fluorescence, two green fluorescent bacterial strains were selected. *S. mutans* UA159*^GFP^* (Deng et al., 2007) and *S. aureus* ATCC 12600*^GFP^* (Li et al., 2011) were grown on Todd Hewitt Broth (THB) (Oxoid, Basingstoke, United Kingdom) and Tryptone Soya Broth (TSB) (Oxoid, Basingstoke, United Kingdom) agar plates, respectively. THB agar plates were supplemented with 10 μg/ml erythromycin (Sigma-Aldrich, St Louis, MO, United States) and TSB agar plates were supplemented with 10 μg/ml tetracycline (Sigma-Aldrich, St. Louis, MO, United States). One colony of *S. mutans* was inoculated in THB supplemented with 10 μg/ml erythromycin and similarly for *S. aureus* in TSB supplemented with 10 μg/ml tetracycline. *S. mutans* UA159*^GFP^* was grown at 37°C with 5% CO_2_ and *S. aureus* was grown under aerobic conditions. After 24 h, these precultures were inoculated in 200 ml (1:20) THB for *S. mutans* and TSB for *S. aureus* without antibiotics and cultured for 16 h at 37°C, yielding early-stationary phase bacteria.

Bacterial cultures were harvested by centrifugation (5 min, 5,000 g, 10°C) and washed twice with a phosphate buffer (2 mM potassium phosphate, 50 mM KCl, and 1 mM CaCl_2_, pH 7.0). After washing, bacteria were resuspended in phosphate buffer with THB or TSB (30:1) to maintain metabolic activity. The bacterial suspension was sonicated (3 × 10 s, 30 W) in an ice-water bath (Vibra Cell Model 375, Sonics and Materials Incorporation, Danbury, CT, United States) and the bacteria were enumerated using a Bürker-Türk counting chamber and suspensions (3 × 10^8^/ml) were diluted in phosphate buffer supplemented with THB or TSB (30:1) to a bacterial concentration, appropriate for the different experiments. All the experiments were done in triplicate with different bacterial cultures.

### Minimal Inhibitory Concentration of Chlorhexidine

A commercial chlorhexidine mouthrinse (Curasept ADS 212, Curasept S.p.A., Saronno, Italy) containing 0.12% (1,200 μg/ml) chlorhexidine digluconate was used in this study. Bacteria were exposed to 2-fold serial dilutions (i.e., 9.6, 4.8, 2.4, 1.2, 0.6, and 0.3 μg/ml) of the mouthrinse in sterile water. 100 μl of different dilutions of the mouthrinse were added to 100 μl of a bacterial suspension (2 × 10^5^/ml) in the appropriate growth medium in 96-well plates and incubated at 37°C for 24 h. After incubation, the minimal inhibitory concentration (MIC) was taken as the lowest chlorhexidine concentration at which no visible growth was observed.

### Development of Chlorhexidine-Resistant Variants

For the development of chlorhexidine-resistant bacterial strains, precultures of *S. mutans* UA159*^GFP^* and *S. aureus* ATCC 12600*^GFP^* were diluted 1:100 in appropriate growth media and grown with chlorhexidine added at its MIC. Bacteria were subcultured daily at the same chlorhexidine concentration over 4 consecutive days. Culturable bacteria that remained present after 4 days were subsequently grown over another sequence of 4 consecutive days at a 0.3 μg/ml higher chlorhexidine concentration than in the previous sequence. MIC was measured daily during each sequence of 4 consecutive days. Subculturing was discontinued when a culture did not demonstrate any bacterial growth by visual inspection of the culture turbidity. In order to check whether prolonged culturing under chlorhexidine exposure affected bacterial fluorescence, fluorescence was monitored regularly on agar plates using an *In Vivo* Imaging System (IVIS, Lumina II, Caliper LifeScience, Hopkinton, MA, United States), with an excitation wavelength of 465 nm and emission in a range from 515 to 575 nm. Cultures after repeated growth in presence of chlorhexidine were stored in a −80°C freezer, with 7% dimethyl sulfoxide added.

### Surface-Enhanced Fluorescence

**FIGURE 1:**
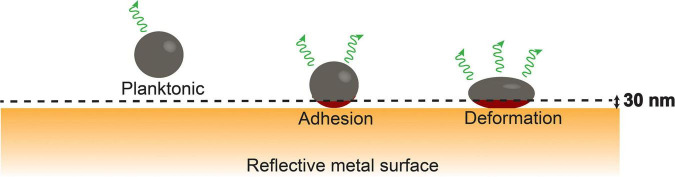
Bacterial cell wall deformation upon adhesion of a fluorescent bacterium to a metal surface from its planktonic state; cell wall deformation will gradually increase under the influence of adhesion forces exerted by the substratum and bring more fluorophores (red color) within the cytoplasm closer to the metal surface within the range of surface enhancement [schematics taken from Carniello et al. (2018a) with permission from the Royal Society of Chemistry].

Molecular fluorescence is enhanced when fluorescent molecules are in the close vicinity (<30 nm) of a metal surface (Lee et al., 2011). This phenomenon, called surface-enhanced fluorescence, can be applied to measure the small deformation that adhering fluorescent bacteria experience in response to the adhesion forces arising from a substratum surface (see Figure 1 for schematics). As a major advantage over atomic force microscopy, surface-enhanced fluorescence is more sensitive and measures over a larger population of bacteria, depending on the number of bacteria adhering to the metal surface.

For surface-enhanced fluorescence, bacteria suspended in phosphate buffer supplemented with growth medium (30:1) were injected in a parallel plate flow chamber, with a glass top-plate and a polished, stainless steel bottom-plate. Fluorescence was measured using a bio-optical imaging system (see above). Images had a field of view of 7.5 cm × 7.5 cm, while exposure time was set at 10 s, employing a focal ratio of 1. Temperature throughout an experiment was maintained at 20°C. With the Living Image software package 3.1 (Caliper LifeScience), a user-defined region of interest was constructed in each image of 4.0 cm × 1.0 cm to calculate the average fluorescence radiance (photons s^–1^ cm^–2^ steradian^–1^).

Background fluorescence in the region of interest was measured in a flow chamber filled with phosphate buffer, supplemented with growth medium (30:1) in the absence of suspended bacteria. This background fluorescence radiance was subtracted from all the fluorescent radiances measured. Next, bacteria were injected into the flow chamber. Sedimentation of suspended bacteria and their adhesion was allowed in the absence of flow under the influence of gravity, while acquiring images every 15 min for 3 h, previously found sufficient for complete sedimentation of all the suspended bacteria (Li et al., 2014). Subsequently, the flow chamber was filled with 5 ml of the commercial chlorhexidine mouthrinse at different dilutions. Fluorescence image acquisition continued every 15 min for an additional 2 h. Phosphate buffer supplemented with growth medium (30:1) was used as a control.

Assuming even intracellular distribution of green fluorescent proteins, the increase in fluorescence radiance due to adhering bacteria relative to planktonic bacteria was expressed as total fluorescence enhancement (Li et al., 2014), according to:


(1) $$TFE\left(t\right)=\frac{R\left(t\right)-R0}{R\left(0\right)-R0}$$

Where, R(t) is the fluorescence radiance at time t, R(0) is the fluorescence radiance measured for a planktonic suspension, and R_0_ is the fluorescence radiance of the background.

### Bacterial Membrane Damage

To determine the percentage of damage upon chlorhexidine exposure to the cell membrane, as an integral part of the cell wall, bacteria were stained with red fluorescent propidium iodide (LIVE/DEAD BacLight Bacterial Viability, Thermo Fisher Scientific, Waltham, MA, United States). Propidium iodide is only able to enter bacteria with a damaged membrane (Lehtinen et al., 2004). Membrane damage was inflicted according to a similar protocol as in surface-enhanced fluorescence. After incubation with chlorhexidine, 15 μl of propidium iodide (20 mM) was added to each well. Fluorescence was imaged using a Leica DM4000B fluorescence microscope with a 40× water objective by taking three images of each well. While closing the red channel, green fluorescent bacteria were enumerated as a measure of the total number of bacteria in an image, while those bacteria also displaying red fluorescence were taken as membrane damaged (Lehtinen et al., 2004).

### Metabolic Activity

The influence of the different concentrations of chlorhexidine on bacterial metabolic activity was determined with 3-(4,5-dimethylthiazol-2-yl)-2,5-diphenyltetrazolium bromide (MTT) reduction assay (Krom et al., 2007). After exposure to chlorhexidine according to a similar protocol as in surface-enhanced fluorescence, MTT solution (0.5 mg/ml thiazolyl blue tetrazolium bromide in phosphate buffer, 10 mg/ml glucose, and 0.1 mM menadione) was added to each well and left for 30 min at 37°C in the dark. After 30 min, wells were washed once with water and mixed with acid isopropanol (5% 1 M HCl in isopropanol) for 15 min. After 15 min, 100 μl of the suspension was removed and added to a new 96-wells plate and absorptions were measured at 560 nm with the FLUOstar Optima Microplate Reader (BMG Labtech, Offenburg, Germany). All the experiments were performed in triplicate with different bacterial cultures. The data were normalized with respect to the metabolic activity of the bacterial strains after 3 h adhesion, i.e., before chlorhexidine exposure.

### Physicochemical Bacterial Cell Surface Characterization

To determine whether the physicochemical bacterial cell surface properties were affected by chlorhexidine exposure, microbial adhesion to hydrocarbons (kinetic MATH assay) and zeta potentials were measured as a function of exposure time of the bacteria to chlorhexidine dissolved in phosphate buffer. MATH was carried out as previously described (Lichtenberg et al., 1985). Briefly, bacteria were resuspended in 3 ml phosphate buffer (pH 7.0) supplemented with chlorhexidine and containing 20:1 hexadecane to an initial absorbance at 600 nm between 0.4 and 0.6. Initial absorbance at time zero ([A0]) was measured photospectrometrically (Spectronic 20 Genesys, Spectronic Instruments, Rochester, NY, United States). After vortexing, the suspension for 10 s, and settling of the bacteria for 10 min, absorbance was measured again at time t ([At]). This procedure was repeated five more times, to allow calculation of the initial rate of bacterial removal from the aqueous phase according to:


(2) $$\text{Rate}\text{of}\text{initial}\text{removal}=\lim_{t\to 0}\frac{\text{d}}{\text{d}t}\log\left(\frac{A_{t}}{A_{0}}\times 100\right)$$

where, t is vortexing time.

The charge properties of the bacterial surfaces were determined by measuring the electrophoretic mobility using a bacterial suspension (3 × 10^8^ bacteria/ml) in phosphate buffer (pH 7.0) supplemented with chlorhexidine. Particulate microelectrophoresis was carried out on a Zetasizer Nano ZS (Malvern Instruments, Worcestershire, United Kingdom). Electrophoretic mobilities measured were converted into zeta potentials, employing the Helmholtz–Smoluchowski equation (Van der Wal et al., 1997).

### Statistical Analysis

Data were statistically analyzed using the paired and two-tailed Student’s *t*-tests with Microsoft Excel 2010. Significance was established at *p* < 0.05.

## Results

### Induction of Chlorhexidine Resistance Upon Subculturing at Different Chlorhexidine Concentrations

Prior to chlorhexidine exposure, the MIC of both the *S. mutans* UA159*^GFP^* and *S. aureus* ATCC 12600*^GFP^* was 1.2 μg/ml. In order to induce chlorhexidine resistance, planktonic cultures of *S. mutans* UA159*^GFP^* and *S. aureus* ATCC 12600*^GFP^* were grown in medium, supplemented with chlorhexidine. After daily subculturing at increasing chlorhexidine concentrations starting at sub-MIC over 4 consecutive days, no culturable *S. mutans* was observed when the chlorhexidine concentration exceeded MIC (Supplementary Figure 1). For *S. aureus*, culturable staphylococci remained present after 13 repetitions of 4 day sequences of growth at stepwise increasing chlorhexidine concentrations, acquiring a MIC of 4.8 μg/ml, i.e., four times the starting MIC of *S. aureus* ATCC 12600*^GFP^* (Supplementary Figure 1). This resistant variant of *S. aureus* ATCC 12600*^GFP^* was subsequently used in further experiments.

### Cell Wall Deformation in Adhering *Staphylococcus aureus* and *Streptococcus mutans* During Chlorhexidine Exposure

**FIGURE 2:**
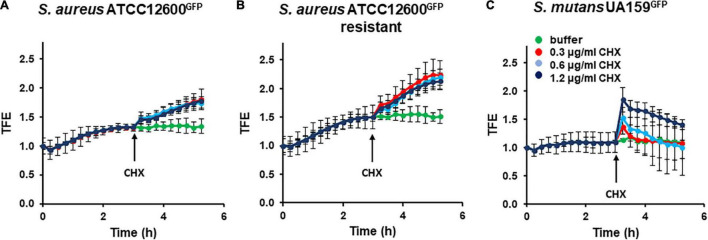
Cell wall deformation derived from total fluorescence enhancement (TFE) in *Staphylococcus aureus* and *Streptococcus mutans* adhering to stainless steel upon exposure to chlorhexidine. Stable cell wall deformation due to adhesion was first established in phosphate buffer supplemented with growth medium (30:1) during 3 h, after which chlorhexidine was added (see arrow). **(A)** TFE of *S. aureus* ATCC 12600*^GFP^* as a function of time for different chlorhexidine concentrations. **(B)** Same as panel a, now for *S. aureus* ATCC 12600*^GFP^*, made resistant to 4.8 μg/ml chlorhexidine. **(C)** Same as panel a, now for *S. mutans* UA159*^GFP^*. All the error bars represent SDs over triplicate measurements with different bacterial cultures.

Both the chlorhexidine-susceptible *S. aureus* strain (Figure 2A) and its chlorhexidine-resistant variant (Figure 2B) demonstrated a gradual increase in total fluorescence enhancement during the first 3 h of sedimentation and adhesion from phosphate buffer to a stainless steel surface, indicative of cell wall deformation. After 3 h, total fluorescence enhancement remained constant for at least 6 h upon continued exposure to buffer. However, the resistant variant (Figure 2B) deformed faster and more extensively than the chlorhexidine-susceptible strain (Figure 2A). Total fluorescence enhancement of *S. mutans* during sedimentation and adhesion followed by continued exposure to phosphate buffer hovered around 1 for at least 6 h, indicating absence of cell wall deformation upon adhesion (Figure 2C). Continued exposure to different concentrations of chlorhexidine after sedimentation and adhesion in phosphate buffer led to a different response in staphylococci than in streptococci. Both the chlorhexidine-susceptible (Figure 2A) and chlorhexidine-resistant *S. aureus* (Figure 2B) demonstrated gradual increase in total fluorescence enhancement that was independent of chlorhexidine concentration. These increases in total fluorescence enhancement remained to increase for at least 6 h, indicative of permanent cell wall deformation. Cell wall deformation was significantly (*p* < 0.05, Student’s *t*-test) larger for the already weakened cell wall of the resistant variant (Figure 2B) as compared with its chlorhexidine-susceptible parent strain (Figure 2A). *S. mutans*, on the other hand, demonstrated a fast increase in total fluorescence enhancement upon chlorhexidine exposure (Figure 2C). The increase in total fluorescence enhancement was dependent on the chlorhexidine concentration during exposure and decreased gradually over time to the level observed in phosphate buffer without chlorhexidine added.

**FIGURE 3:**
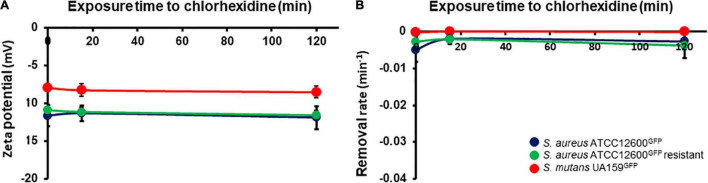
Physicochemical cell surface properties of *S. aureus* and *S. mutans* upon exposure to chlorhexidine. **(A)** Zeta potentials of bacteria in phosphate buffer as a function of time during exposure to 1× minimal inhibitory concentration (MIC) chlorhexidine. **(B)** Initial removal rates of bacteria from phosphate buffer by hexadecane as a function of time during exposure to 1× MIC chlorhexidine. All the error bars represent SDs over triplicate measurements with different bacterial cultures.

The physicochemical surface properties, bacterial zeta potentials (Figure 3A), and hydrophobicities (Figure 3B) relevant for adhesion did not change upon chlorhexidine exposure for all the three strains.

### Cell Wall Damage in Adhering *Staphylococcus aureus* and *Streptococcus mutans* Measured Using Fluorescence Staining

Cell wall weakening was further studied by assessing cell wall damage based on cell membrane permeability to red fluorescent propidium iodide. Damage to the membrane, as an integral part of the cell wall, can be demonstrated at microscopic level and over a large population of bacteria by staining with red fluorescent propidium iodide, entering only damaged cell walls with increased membrane permeability.

**FIGURE 4:**
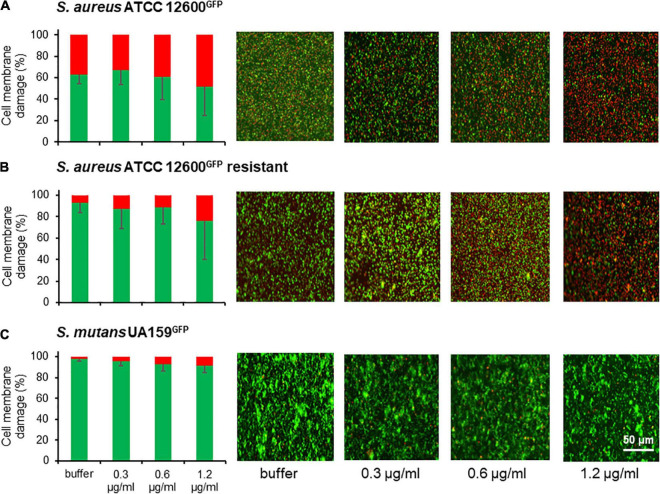
Cell wall weakening as a result of membrane damage of *S. aureus* and *S. mutans* after 2 h exposure to chlorhexidine. **(A)** Percentage cell membrane damaged bacteria after exposure to different chlorhexidine concentrations for *S. aureus* ATCC 12600*^GFP^*. Cell membrane damage was inferred from red fluorescence after staining with propidium iodide. Total fluorescence prior to exposure to chlorhexidine was taken as 100%. **(B)** Same as panel a, now for *S. aureus* ATCC 12600*^GFP^*, made resistant to 4.8 μg/ml chlorhexidine. **(C)** Same as panel a, now for *S. mutans* UA159*^GFP^*. All the error bars represent SDs over triplicate measurements with different bacterial cultures.

Exposure of adhering bacteria to different concentrations of chlorhexidine caused nearly 50% of *S. aureus* to become membrane damaged, irrespective of concentration (Figure 4A). In the chlorhexidine-resistant *S. aureus* variant, exposure to chlorhexidine led to significantly fewer bacteria with membrane damage (<25%) (Figure 4B). Very few *S. mutans* became membrane damaged upon exposure to chlorhexidine (Figure 4C). Collectively, these data suggest that *S. mutans* suffers considerably less cell wall damage upon exposure to chlorhexidine than the susceptible *S. aureus* strain (*p* < 0.05) and even less than a chlorhexidine-resistant *S. aureus* variant although initially upon exposure to chlorhexidine it deforms equally as staphylococci do.

### Self-Repair of Cell Wall Damage Inflicted by Chlorhexidine

**FIGURE 5:**
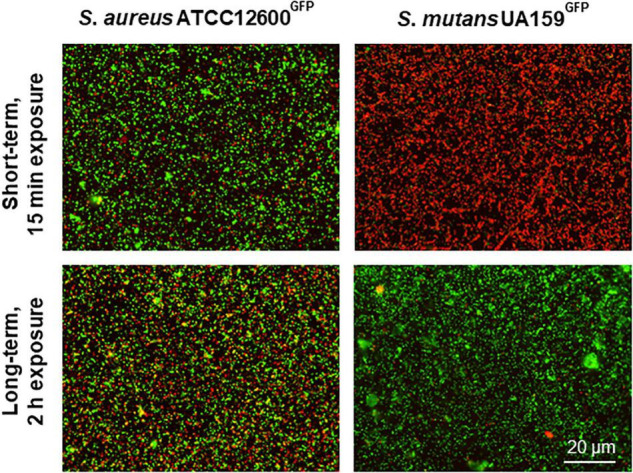
Self-repair in cell membrane damaged *S. aureus* and *S. mutans* upon 15 min or 2 h exposure to chlorhexidine. Cell membrane damaged bacteria after 15 min exposure to 1.2 μg/ml chlorhexidine and after 2 h exposure to 1.2 μg/ml chlorhexidine. Both the strains are green fluorescent, while red fluorescent bacteria are cell wall damaged as inferred from the penetration of red fluorescent propidium iodide through the damaged cell wall.

In order to verify the suggestion of self-repair, cell wall damage was inflicted upon both the strains by a short-term, 15 min exposure to 1.2 μg/ml chlorhexidine (MIC) and compared with the cell wall damages inflicted by longer-term, 2 h exposure. Short-term exposure to chlorhexidine yielded only 10% of the *S. aureus* bacteria to become cell wall damaged, while 99% of *S. mutans* was already cell wall damaged, allowing penetration of red fluorescent propidium iodide (Figure 5). Upon continued exposure to chlorhexidine up to 2 h, however, 38% of the *S. aureus* appeared cell wall damaged than upon short-term exposure, but almost the entire population (96%) of *S. mutans* within a microscopic field of view had fully self-repaired the damage over time. A sub-MIC concentration of chlorhexidine showed similar self-repair as observed at MIC (Supplementary Figure 2). This confirms the presence of a self-repair mechanism against cell wall damage inflicted by chlorhexidine in *S. mutans* that is absent in *S. aureus*. However, at chlorhexidine concentration far above MIC (600 μg/ml), self-repair in *S. mutans* was no longer observed (see also Supplementary Figure 2).

### Metabolic Activity During Chlorhexidine Exposure to Adhering *Staphylococcus aureus* and *Streptococcus mutans*

**FIGURE 6:**
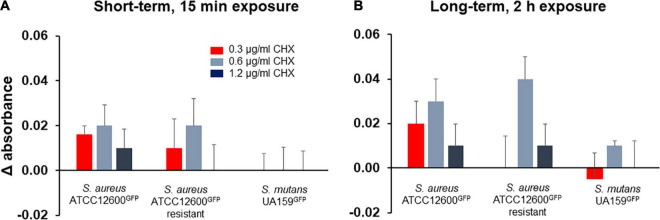
Metabolic activity of adhering *S. aureus* and *S. mutans* upon growth in presence of different concentrations of chlorhexidine, expressed with respect to the metabolic activity measured just prior to chlorhexidine exposure. **(A)** Metabolic activity of the bacterial strains after exposure to chlorhexidine for 15 min. **(B)** Metabolic activity of the bacterial strains after exposure to chlorhexidine for 2 h. All the error bars represent SDs over triplicate measurements with different bacterial cultures.

Initially, short-term (15 min) exposure to 1.2 μg/ml chlorhexidine did not show an increase in metabolic activity (Figure 6) in the resistant *S. aureus* and in *S. mutans*, but a small increase in the susceptible *S. aureus*. Exposure to the lower chlorhexidine concentrations shows a small, but not significant increase in metabolic activity for the *S. aureus* strains as compared to buffer exposure. After 2 h exposure to chlorhexidine, the metabolic activity of both the *S. aureus* strains at 0.6 and 1.2 μg/ml was significantly (*p* < 0.05, Student’s *t*-test) higher than of the *S. mutans* strain. *S. mutans* exhibited no significant increased metabolic activity upon self-repair of chlorhexidine-inflicted cell wall damage (Figure 6).

## Discussion

In this article, we speculated upon the question of why *S. aureus* strains can be found resistant to chlorhexidine, while other bacteria, most notably oral *S. mutans*, largely remain susceptible to chlorhexidine. The data presented here are in part derived from the measurement of cell wall deformation using surface-enhanced fluorescence, providing a never explored pathway to understand the different responses of *S. aureus* and *S*. *mutans* toward chlorhexidine. Collectively, the data indicate that chlorhexidine at low concentration (1.2 μg/ml), characteristic for the substantive release of chlorhexidine adsorbed to oral tissues (Kumar, 2017; Reda et al., 2021), causes irreparable damage to the *S. aureus* cell wall. At the same time, cell wall damage causes increased efflux of chlorhexidine, impeding the buildup of a high chlorhexidine concentration in the cytoplasm that might cause precipitation of proteins and nucleic acids. This efflux is accompanied by increased metabolic activity, suggesting activation of efflux pumps that may be involved in the development of chlorhexidine resistance in *S. aureus* (Costa et al., 2013; LaBreck et al., 2020). Exposure of *S. mutans* to chlorhexidine also yields cell wall damage that occurs on one hand, much faster than in *S. aureus*, but at the other hand is also rapidly self-repaired after exposure to chlorhexidine. As a result of this rapid self-repair, efflux of chlorhexidine is impossible leading to solidification of the cytoplasm (McDonnell and Russell, 1999) due to precipitation of proteins and nucleic acids. Therewith, self-repair becomes suicidal and prevents the development of a chlorhexidine-resistant progeny in *S. mutans*. In line, no increased metabolic activity was observed in *S. mutans* upon exposure to chlorhexidine. Importantly, the physicochemical surface properties relevant for adhesion (Figure 3) did not change upon chlorhexidine exposure for all the three strains and cannot be held accountable for the changes in cell wall deformation observed.

*S. aureus* and *S. mutans* are both the Gram-positive bacterial strains and both their cell walls are composed of an array of surface appendages, a relatively thick layer of peptidoglycan, and a lipid membrane. The relatively rigid peptidoglycan layer is designed to resist intracellular pressure and maintain cell shape (Dover et al., 2015). Cell shape is compromised by the adhesion forces a bacterium experiences when it adheres to a surface, which leads to nanoscopic deformation of the cell wall. When the adhesion force experienced equals the opposing elastic forces from the peptidoglycan layer and the intracellular pressure, cell wall deformation stabilizes (see Figure 1), as observed here (see Figure 2). Upon exposure to chlorhexidine, cell wall deformation increases in both the strains, indicative of cell wall damage and leakage of intracellular material (Gilbert and Moore, 2005) and therewith, a loss of intracellular pressure, leading to ongoing deformation unless the cell wall damage is rapidly self-repaired, as concluded here to occur in *S. mutans* demonstrating reversible cell wall deformation after chlorhexidine exposure (Figure 2C).

We here used surface-enhanced fluorescence as a measure for cell wall deformation. As an advantage of surface-enhanced fluorescence over microscopic (fluorescence based) techniques, it measures cell wall deformation over several millions of bacteria, while microscopic techniques yield visualization of the damage of only a couple of hundred bacteria at best, depending on the type of microscopy and magnification applied. Here, we employed fluorescence microscopy using propidium iodide to demonstrate that increased cell wall deformation included increased permeability of the cell membrane. The bacterial cell membrane is an integral part of the cell wall and becomes penetrable to propidium iodide when damaged (Lehtinen et al., 2004). Chlorhexidine is a positively-charged molecule known to damage the cell wall and destabilize the membrane by interacting with negatively-charged phospholipids to create membrane holes (McDonnell and Russell, 1999). Microscopic holes after chlorhexidine exposure have been found in both the Gram-positive and Gram-negative bacteria. The number of holes increased with exposure time to chlorhexidine and concentration (Cheung et al., 2012).

Once chlorhexidine-induced cell wall deformation has commenced, this can lead to a cascade of different events. Bacterial cell wall deformation enhances the surface area of the lipid bilayer membrane enveloping the cytoplasm. This affects the lipid bilayer tension in the membrane that can subsequently activate mechanosensitive proteins located in the cell wall (Harapanahalli et al., 2015; Laventie and Jenal, 2020). In *S. aureus*, mechanosensing due to cell wall deformation has been demonstrated to be involved in the gating of mechanosensitive transmembrane channels (Carniello et al., 2020) to regulate transport across the membrane and the activation of nisin efflux pumps (Carniello et al., 2018b). Clearly, this cascade of events in *S. aureus* is energy-consuming, as evidenced by the increased metabolic activity of *S. aureus* in presence of chlorhexidine (see Figure 6). Also, streptococcal membrane proteins have been suggested to be involved in membrane pumping (Lorca et al., 2007), but their possible activation by cell wall deformation has not yet been demonstrated. Likely, the membrane insertases YidC–Oxa1–Alb3 that function as signaling proteins and catalyze the synthesis of new membrane proteins are responsible for the rapid self-repair of cell wall damage inflicted by chlorhexidine in *S. mutans* (Suntharalingam et al., 2009; Dalbey et al., 2014). *S. mutans* has two YidCs of which YidC1 preferentially operates in the absence of signal recognition particle pathways and YidC2 being the preferred insertase in presence of signal recognition particle pathways (Vasquez et al., 2021). Possession of two YidC paralogs may be the reason for the rapid self-repair under chlorhexidine stress in *S. mutans* as compared with *S. aureus* possessing only the YidC2 paralog (Shahmirzadi et al., 2016). Self-repair impedes reduction of intracellular chlorhexidine concentration that may lead to lethal solidification of the cytoplasm. Simultaneous self-repair and death due to cytoplasm solidification (McDonnell and Russell, 1999; Cieplik et al., 2019) explain the low metabolic activity in *S. mutans* during exposure to chlorhexidine (see also Figure 6).

Subculturing *in vitro* of *S. mutans* in the presence of increasing concentrations of chlorhexidine up to 1.5 μg/ml can increase the MIC of *S. mutans* up to 3-fold to yield a more resistant variant (Kaspar et al., 2019). We, oppositely, found that repeated subculturing at 1.2 μg/ml and stepwise higher could not induce resistance. These two opposing results must be viewed with respect to the clinical observation that *S. mutans* resistance to chlorhexidine is extremely rare (Cieplik et al., 2019). Chlorhexidine does not distinguish itself from other oral antimicrobials such as cetylpyridinium chloride in its ability to kill bacteria at high concentrations (Renton-Harper et al., 1996). Rather, the ability of chlorhexidine to adsorb to oral tissues and its subsequent release over prolonged periods of time up to 12 h is the key to its success as a most effective oral antimicrobial. Prolonged release in the oral cavity after a 30–60 s rinse yields concentrations of chlorhexidine that critically varies above and below the concentrations applied in the study by Kaspar et al. (2019) and ours. This suggests that the response of *S. mutans* to chlorhexidine may critically depend on concentration (Cieplik et al., 2019). Although chlorhexidine concentrations in saliva hover around the concentrations used by Kaspar et al. (2019) and ours, it is likely that *S. mutans* encounters higher chlorhexidine concentrations than occurring in saliva as it adheres to salivary conditioning films that constitute a reservoir of absorbed and adsorbed chlorhexidine. Suicidal self-repair would, therewith, explain why chlorhexidine-resistant *S. mutans* have not been clinically found.

This study presents a new perspective on the question why clinically *S. mutans* has hardly shown any signs of resistance to chlorhexidine by comparing its response to chlorhexidine with the response of *S. aureus*. Suicidal self-repair of cell wall damage upon chlorhexidine exposure in *S. mutans* is essential in maintaining its susceptibility for chlorhexidine during the periods that low concentrations of chlorhexidine exist in the oral cavity. *S. aureus* lacks such an overly effective self-repair mechanisms and found ways for effective efflux of chlorhexidine that allowed it to develop chlorhexidine resistance.

## Data Availability Statement

The original contributions presented in the study are included in the article/Supplementary Material, further inquiries can be directed to the corresponding author.

## Author Contributions

ML and VS contributed to conception, design, data acquisition, analysis and interpretation, and drafted and critically revised the manuscript. GG-D, MD, and WW contributed to data acquisition, analysis and interpretation, and manuscript reviewing. VC contributed to data acquisition and critically revised the manuscript. HB, HM, and YR contributed to conception, design, data analysis and interpretation, and drafted and critically revised the manuscript. All authors gave final approval and agreed to be accountable for all the aspects of this study.

## Author Disclaimer

Opinions and assertions contained herein are those of the authors and are not construed as necessarily representing views of the funding organization or their respective employer(s).

## Conflict of Interest

HB was also director of a consulting company SASA BV, Thesinge, Netherlands. The remaining authors declare that the research was conducted in the absence of any commercial or financial relationships that could be construed as a potential conflict of interest.

## Publisher’s Note

All claims expressed in this article are solely those of the authors and do not necessarily represent those of their affiliated organizations, or those of the publisher, the editors and the reviewers. Any product that may be evaluated in this article, or claim that may be made by its manufacturer, is not guaranteed or endorsed by the publisher.
