# Supplementary material for: A Comparison of the Adaptive Response of Staphylococcus aureus vs. Streptococcus mutans and the Development of Chlorhexidine Resistance
Source: Front Microbiol. 2022 May 19;13:861890. doi: 10.3389/fmicb.2022.861890 (PMC9186159; doi:10.3389/fmicb.2022.861890)
Supplement: Supplementary file 1 [file Presentation_1.pdf]

frontiers

Supplementary Material

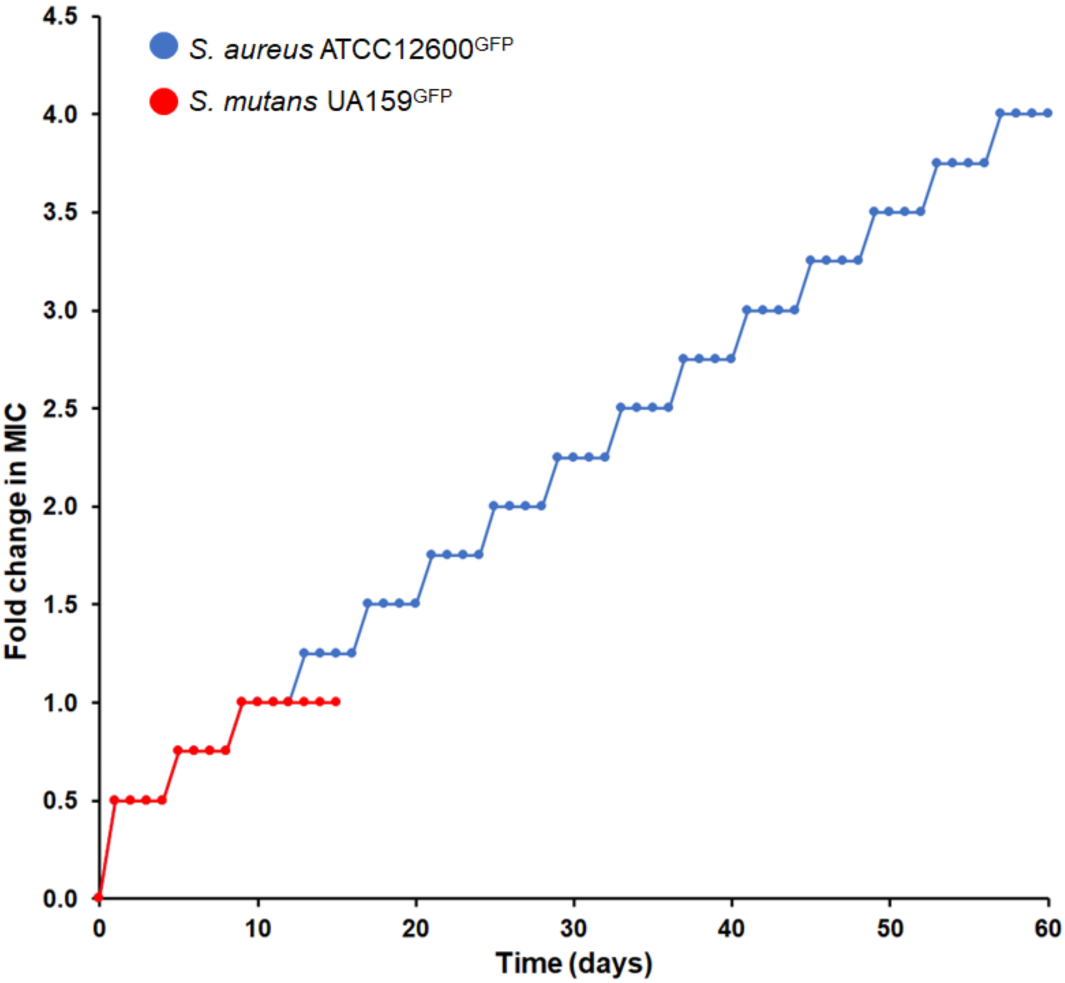

Supplementary Figure S1. Development of chlorhexidine resistance in *S. aureus* ATCC12600^{GFP} and *S. mutans* UA159^{GFP} as a function of the number of days sub-cultured in the presence of increasing concentrations of chlorhexidine. Chlorhexidine resistance was expressed as a fold change in MIC (1.2 µg/ml chlorhexidine for both strains). Bacteria were sub-cultured daily at the same chlorhexidine concentration over 4 consecutive days at 0.3 µg/ml higher chlorhexidine concentrations, starting at a sub-MIC concentration of 0.3 µg/ml chlorhexidine. Sub-culturing was stopped, when bacterial growth was absent, as observed by visual inspection of the culture turbidity.

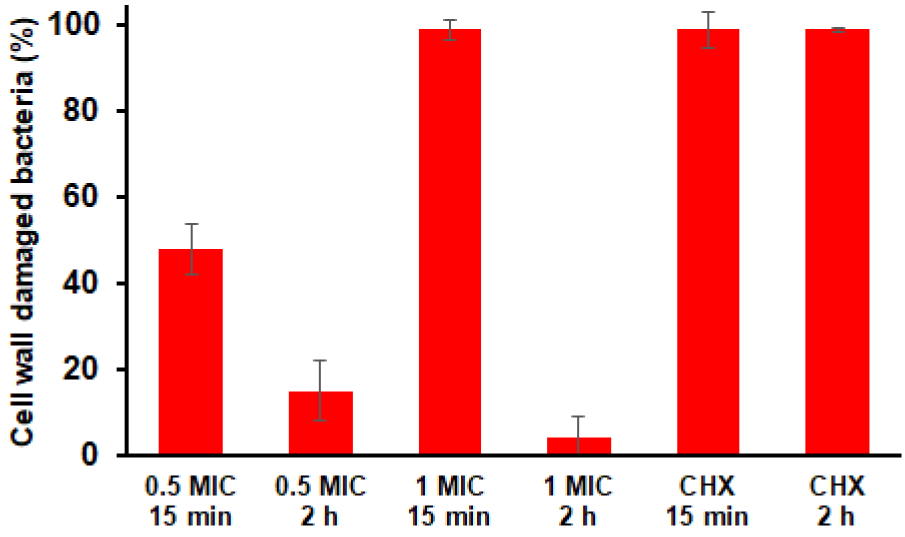

Supplementary Figure 2. Cell membrane damage and time-dependent self-repair of membrane damage in *Streptococcus mutans* UA159^{GFP} upon 15 min and 2 h exposure to chlorhexidine (CHX). The percentage cell membrane damaged bacteria after 15 min and 2 h exposure to a sub-MIC of chlorhexidine (0.6 µg/ml), MIC (1.2 µg/ml) and a two-fold diluted chlorhexidine mouthrinse (600 µg/ml) was expressed with respect to unexposed streptococci.
